# Study on the Hydrogen Embrittlement of Nanograined Materials with Different Grain Sizes by Atomistic Simulation

**DOI:** 10.3390/ma15134589

**Published:** 2022-06-29

**Authors:** Jiaqing Li, Ziyue Wu, Fang Wang, Liang Zhang, Chilou Zhou, Cheng Lu, Lin Teng, Qifeng Lin

**Affiliations:** 1 College of Chemical Engineering, Fuzhou University, Fuzhou 350116, China; jiaqing@fzu.edu.cn (J.L.); 210427154@fzu.edu.cn (Z.W.); 2 Fujian Special Equipment Inspection and Research Institute, Fuzhou 350008, China; wangfang2080@163.com; 3 International Joint Laboratory for Light Alloys (MOE), College of Materials Science and Engineering, Chongqing University, Chongqing 400044, China; liangz@cqu.edu.cn; 4 School of Mechanical and Automotive Engineering, South China University of Technology, Guangzhou 510641, China; mezcl@scut.edu.cn; 5 School of Mechanical, Materials, Mechatronic and Biomedical Engineering, University of Wollongong, Wollongong, NSW 2522, Australia; chenglu@uow.edu.au; 6 College of Computer and Data Science, Fuzhou University, Fuzhou 350116, China

**Keywords:** hydrogen embrittlement, nanograined materials, intergranular deformation, brittle fracture, grain refinement

## Abstract

Although hydrogen embrittlement (HE) behavior has been extensively studied in bulk materials, little is known about H-related deformation and the fracture of nanograined materials. In this study, H segregation and HE mechanisms of nanograined Fe with different grain sizes are unveiled, following the employment of classical molecular dynamics simulations. The H segregation ratio increased, but the local H concentration at the grain boundaries (GBs) decreased with decreases in the grain size at a given bulk H concentration. The results demonstrate that H atoms increased the yield stress of nanograined models irrespective of the grain size. Furthermore, it is revealed that brittle fractures were inhibited, and the resistance to HE increased as the grain size decreased, due to the fact that the small-grain models had a lower local H concentration at the GBs and an enhanced GB-mediated intergranular deformation. These results are a clear indication of the utility of grain refinement to resist H-induced brittle failure.

## 1. Introduction

As a renewable energy source, hydrogen gas will be widely used in transportation, energy storage, industry, construction and other fields in the near future [1,2]. The global market of hydrogen energy is now about 125 billion US dollars and has the potential to double to 250 billion US dollars by 2030 and exceed 1 trillion US dollars [3,4]. Safe and economical hydrogen storage and transportation is the key to the practical and industrial utilization of hydrogen energy. However, hydrogen storage and transportation systems such as pipelines, long-tube trailers and hydrogen refueling stations operate under high-pressure and high-purity hydrogen environments for a long time, which usually leads to the ‘hydrogen embrittlement’ (HE) phenomenon [5,6]. As the name suggests, HE causes a loss in ductility, an increase in the fatigue crack growth rate and often includes sudden brittle failure and fractures [7,8,9]. Over the years, safety accidents related to HE problems have been widely reported and have led to serious casualties and property losses.

Under various service environments, H has the ability to absorb into metallic materials and diffuse through materials due to its small size. These diffusible H atoms are more prone to be trapped by microstructural features such as vacancies [10,11], dislocations [12,13], grain boundaries (GBs) [14,15], crack tips [16,17] and precipitates [15,18]. Extensive experimental studies have revealed that H-related failure is often cleavage-like and intergranular, underlining the importance of GBs in the HE of polycrystalline metals [19,20,21,22]. Various GB-related HE theories have been proposed and developed. One is H-enhanced decohesion (HEDE), where interstitial H weakens the cohesive strength of GBs by the dilatation of the atomic lattice [23,24,25,26]. However, HEDE alone cannot explain the observed strong plasticity activities beneath H-induced quasi-cleavage and intergranular facets. H-enhanced localized plasticity (HELP) posits that H promotes dislocation plasticity and dislocation–GB interactions [27,28,29,30], which leads to material softening. This mechanism has been proposed as crucial for the creation of the environment for the activation of intergranular failure [20,31].

With the importance of GBs in H-related failure, increasing the GB density via grain refinement is anticipated to affect the susceptibility of materials to intergranular HE. Severe plastic deformation (SPD) techniques, such as high-pressure torsion (HPT) [32,33,34,35,36,37], accumulative roll bonding (ARB) [38,39,40], equal channel angular pressing (ECAP) [41,42,43] and dynamic plastic deformation (DPD) [44] have been widely applied to achieve grain refinement, modify the texture and improve the mechanical properties of various metallic materials. In comparison with other SPD techniques, HPT is much more efficient in refining the microstructure and improving the mechanical strength. Besides, the HE performance of SPD-processed materials has attracted much research interest. For example, recently, Mine et al. [32] conducted HPT and subsequent annealing to produce ultrafine-grained-type 304 steel and found that ultrafine-grained specimens reduced the HE susceptibility and showed a good combination of strength and ductility. Fang et al. [44] developed a nanotwinned 304 austenite stainless steel through the DPD technique, pointing out that the DPD-annealed sample with 41% nanotwins showed a significantly high HE resistance. Although these experiments elucidated the effect of grain size on HE sensitivity, samples were usually on a micrometer scale. When grain size is decreased to a nanometer scale and a critical grain size where the GB role is more prominent, the GB-related HE fracture of nanograined materials should be alien from that of the ultrafine and coarse-grain counterparts. Unfortunately, due to the limitations of current experimental techniques on determining the nanoscale distribution of H atoms, little is known about the effect of H on nanograined materials.

Considering that atomistic simulation is capable of identifying the nanoscale H trapping at GBs [14,19,26,29,45,46,47] and quantifying the plasticity activities of nanograined materials [48,49,50,51], it is employed here to probe the HE of nanograined materials, with the emphasis on the influence of grain size on H segregation, H-related deformation and H-induced fracture. As steels are more susceptible to HE, α-Fe was selected as the model material. The present study is organized as follows: The details of the simulation setup are described in Section 2. H segregation and deformation mechanisms of nanograined Fe models without and with solute H are given in Section 3. The simulation results are discussed in Section 4, followed by the main conclusions in Section 5.

## 2. Materials and Methods

All molecular dynamics (MD) simulations were carried out by the open-source Large-scale Atomic/Molecular Massively Parallel Simulator (LAMMPS) [52] with the Finnis–Sinclair-type embedded atom method potential for Fe-H [28]. The initial samples were geometrically established via the Voronoi program. Periodic boundary conditions were prescribed along all three directions. A total of 8 nanograined Fe models with different in-plane (x-y plane) grain diameters of 4 nm, 6 nm, 8 nm, 10 nm, 12 nm, 14 nm, 16 nm and 18 nm were considered, and denoted as NG_d_ with d being the grain size. The initial configurations were optimized by performing the conjugate gradient energy minimization technique. Crystal structures were calculated by common neighbor analysis (CNA) and centro-symmetry parameter (CSP), and dislocation plasticity was analyzed by the dislocation extraction algorithm (DXA) using the Open Visualization Tool (OVITO) [53], in which output data generated in MD simulations, atomistic Monte-Carlo and other particle-based simulations can be visualized and analyzed. For an investigation of the HE of nanograined Fe, models filled with H atoms, which corresponded to bulk H concentrations (C_0_) of 0.25%, 0.5%, 0.75% and 1%, were prepared. After the insertion of H atoms, the created models were first relaxed at 700 K for 1 ns and were then cooled down at a temperature of 300 K for 1 ns, followed by a further relaxation at 300 K for 3 ns [54]. It was assumed that the heating process can bring boundary structure and H segregation into a state of equilibrium. Uniaxial tension was conducted by stretching the simulation boxes along the x direction while keeping the y and z directions stress free under the isothermal–isobaric ensemble (NPT). The temperature of the whole system was maintained at 300 K using the Nose–Hoover thermostat [55]. Two different timesteps of 0.5 fs and 1 fs were used for the models in the presence and absence of H, respectively [16]. It is worth noting that the applied tensile strain rate was set at 5 × 10^8^/s, which is much higher by several orders of magnitude than that in real experiments. Under such a high strain rate, the H diffusion process over the time scales of loading is typically not captured. However, this is an inherent limitation of the MD method, and it will be presented that the influence of H atoms on the response of nanograined Fe with different sizes is in line with experimental observations of the H-induced failure.

## 3. Results

H segregation in metals has a profound effect on mechanical properties and ultimate failure. In the following, H segregation at the GBs of nanograined Fe models is presented in Section 3.1, and its influence on the mechanical behavior, deformation mechanisms and fracture response of nanograined Fe models with different grain sizes is given in Section 3.2 and Section 3.3.

### 3.1. GB Structures and H Segregation in Nanograined Fe Models

**Figure 1:**
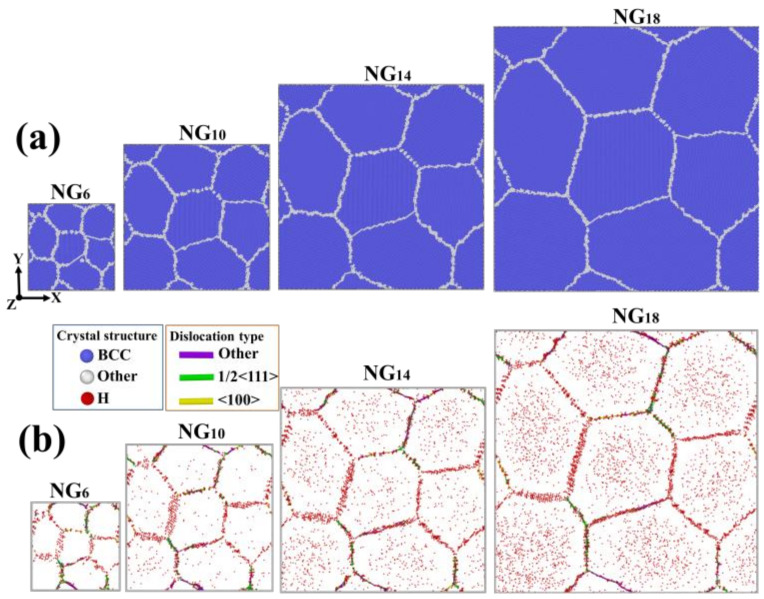
(**a**) Atomic configurations of nanograined Fe models with various grain sizes; (**b**) H distribution in nanograined models. Atoms are colored according to CNA, where Fe atoms with a perfect structure are colored blue, atoms at GBs are colored white and H atoms are colored red. Possible dislocations are indicated by their types.

The equilibrated structures of nanograined Fe are shown in Figure 1a. The considered GBs in nanograined models are characterized by the GB atom ratio and the GB free volume. The GB atom ratio can be obtained by the number of GB atoms against the total number of Fe atoms, while the GB free volume is defined as:

(1) $$\Delta V_{\mathrm{GB}}=\frac{V_{\mathrm{nano}}-N_{\mathrm{Fe}}V_{\mathrm{atom}}}{N_{\mathrm{Fe}}V_{\mathrm{atom}}}$$

where $V_{\mathrm{nano}}$ is the volume of the nanograined model, $N_{\mathrm{Fe}}$ means the total number of Fe atoms and $V_{\mathrm{atom}}$ is the volume of one perfect Fe atom.

**Figure 2:**
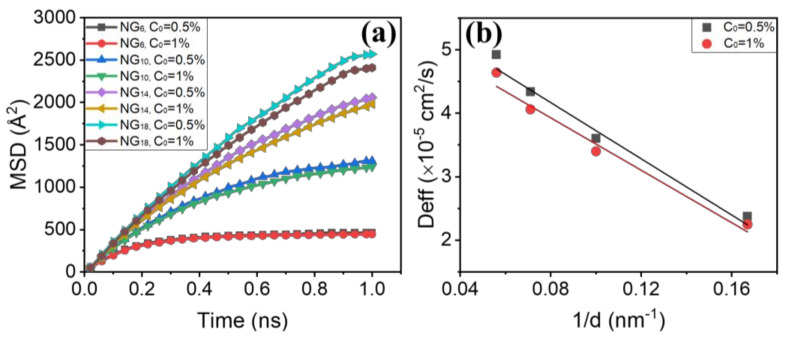
(**a**) MSD of H atoms in models with different grain sizes vs. time; (**b**) $D_{eff}$ as a function of 1/d.

To investigate H segregation in nanograined Fe models, H atoms were added into models and diffused into GBs. The H distribution in four models is depicted in Figure 1b. It is clear that most of the H atoms were trapped at GBs and dislocation cores, while other H atoms occupied bulk sites. During the H diffusion process, the H diffusion coefficient can be calculated as [56]:

(2) $$D_{eff}=lim_{t\to \infty }\;\frac{1}{6N}\sum_{i=1}^{N}{\left(\vec{r_{i}}\left(t\right)-\vec{r_{i}}\left(t_{0}\right)\right)}^{2}$$

where $\frac{1}{N}\sum_{i=1}^{N}{\left(\vec{r_{i}}\left(t\right)-\vec{r_{i}}\left(t_{0}\right)\right)}^{2}$ defines the mean-squared displacement (MSD) of H atoms. N is the total number of H atoms, $\vec{r_{i}}\left(t_{0}\right)$ is the original position of the *i*th H atom and $\vec{r_{i}}\left(t\right)$ is the position of the *i*th H atom at time t. The MSD curves vs. simulation time are shown in Figure 2a, in which H diffusion velocity (the slope of plots) was initially high, and then decreased as the H atoms arrived at the GBs. Taking the NG_6_ as an example, after 0.2 ns H atoms were trapped by the GBs, and the H diffusion became relatively low due to the GB trapping effect [54]. With the increase in grain size the GB density decreased and the GB trapping effect consequently weakened, rendering fast H diffusion. The H diffusion coefficients calculated from our simulations are shown in Figure 2b and are compared with the available experimental values [57,58]. It is interesting to note that the H diffusion at a lower concentration (C_0_ = 0.5%) was found to be higher than that at a higher concentration (C_0_ = 1%). This confirms that the H diffusion process can be influenced by other H interstitials.

After the H diffusion process, the H atoms segregating at the GBs were counted and divided by total number of H atoms to obtain the segregation ratio (f_seg_). The corresponding values are tabulated in Table 1. For the nanograined models, f_seg_ was almost insensitive to C_0_. The reason can be attributed to the fact that in an equilibrated state, f_seg_ depends on the number of trapping sites that GBs provide; for the same model, the trapping sites were constant and f_seg_ was therefore unchanged. Furthermore, it can be seen that f_seg_ decreased with increases in the grain size, which is indicative of the fact that small-grain models have a stronger ability to trap H atoms. This observation can be linked with the GB atom ratio and $\Delta V_{\mathrm{GB}}$; small-grain models have higher values of the GB atom ratio and $\Delta V_{\mathrm{GB}}$, suggesting that more trapping sites can accommodate H atoms.

The local H concentration at GBs (C_H-GB_) is calculated as the number of H atoms at GBs divided by the boundary area. Table 1 shows that the local H concentration increased with increases in the grain size. Due to the grain refinement, small-grain models have a higher segregation ratio, but a higher GB density at the same time. As a result of the ‘dilution effect’, the C_H-GB_ of small-grain models is lower than that of large-grain ones, evidenced by that the C_H-GB_ of NG_6_ was only half of that of NG_18_.

### 3.2. Mechanical Behavior and Deformation Mechanisms of Nanograined Fe Models without H

**Figure 3:**
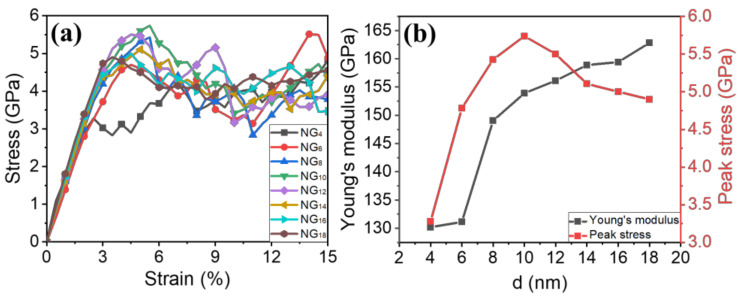
(**a**) Tensile stress–strain relationships of nanograined Fe cases with various grain sizes; (**b**) Young’s modulus and peak stress as a function of d.

The stress–strain relationships of nanograined Fe cases with varying grain sizes are plotted in Figure 3. The tensile stress is calculated by dividing the stress tensor of the entire system over the volume of the system, and tensile strain is obtained from the applied strain rate multiplied by the deformation time. It is clear from the slope of stress–strain relationships that Young’s modulus was reduced with decreases in the grain size. This phenomenon may be associated with the fact that models with smaller grain sizes have a higher GB fraction, which promotes the intergranular deformation rather than intragranular deformation, leading to a lower Young’s modulus. On the other hand, Figure 3b clearly shows that the peak stress increased as the grain size decreased to 10 nm, but a reverse relation occurred with a further reduction in the grain size, namely ‘inverse’ Hall–Petch behavior.

**Figure 4:**
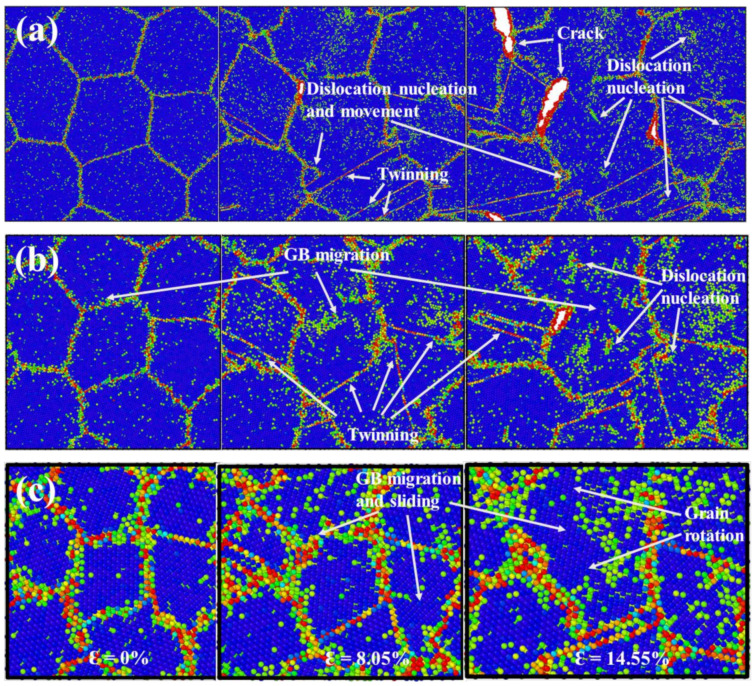
(**a**–**c**) The atomic snapshots of NG_18_, NG_10_ and NG_6_ at different tensile strains under tension, respectively. Images are colored by CSP, and various deformation mechanisms are indicated by arrows.

Figure 4 presents the atomic configurations of the models with grain sizes of 18 nm, 10 nm and 6 nm, respectively. In the case of NG_18_, dislocation-dominated plasticity was the main deformation mechanism, as seen in Figure 4a. Heterogeneous dislocation nucleation and emission from the GB occurred at a strain of ε = 8.05%, which is in accordance with our previous findings that GBs are the dislocation source [23,29]. Besides, dislocations were also generated from the grain interior in several locations. These intragranular dislocation mechanisms dominated the plastic deformation processes. Deformation twins only occurred at some certain grains and remained almost unchanged with further applied tensile strain, indicating that these twins play a secondary role in the deformation process of the NG_18_ model.

Figure 4b shows the atomic snapshots of the NG_10_ model in the absence of H. It is clear that the GB-mediated process was increasingly more important, as the GB structures became disordered during the deformation process. GB migration and grain growth occurred at a strain of ε = 8.05% with two separated grains growing into one large grain. On the other hand, there were more deformation twins in the NG_10_ model than NG_18_ model. These twins nucleated from the GBs by a continuous emission of partials and evolved in the grain interior. Previous reports of nanocrystalline Mo and gold films pointed out that nanotwins not only produced plastic strain and intergranular failure [59], but also assisted grain coarsening by changing the local grain misorientation and mobilizing the GB during the plastic deformation process [60]. Our results further confirm that these twins are important deformation carriers in nanograined Fe materials and induce the grain rotation and growth. Next, the intergranular deformation of the NG_6_ model is examined and shown in Figure 4c. Under tension, most of grains evolved from their initial shapes, and a majority of GBs migrated from their original positions. The occurrence of GB sliding, and migration accompanied by grain rotation and growth suggests that GB-mediated deformation is the dominating deformation mechanism of the NG_6_ model. It is worth noting that there were no dislocation nucleation or dislocation pile-up events, unlike the cases of NG_18_ and NG_10_.

The aforementioned deformation process indicates a change in the deformation mechanisms from intragranular mode to intergranular mode. When the grain size was above the 10 nm, nanograined models deformed primarily by the intragranular mode. Specifically, full dislocations nucleated from the grain interior and GBs, and further dislocation emission and movement occurred. However, when the grain size was less than 10 nm, the dominant deformation mechanism of nanograined Fe was intergranular deformation. The transition can be associated with the fact that the decreased grain size resulted in the increase in the GB atom ratio, as tabulated in Table 1. The GB atom ratio of NG_18_ was 5.51%, while the value increased to 14.71% in the case of NG_6_. The higher GB atom ratio and GB density encouraged the GB-mediated deformation processes such as GB sliding and migration. The discrepancy of deformation mechanisms of nanograined Fe models suggests that HE mechanisms should be dependent on the grain size, which will be discussed in the following paragraphs.

### 3.3. Deformation Mechanisms and the Fracture Response of Nanograined Fe Models with H

**Figure 5:**
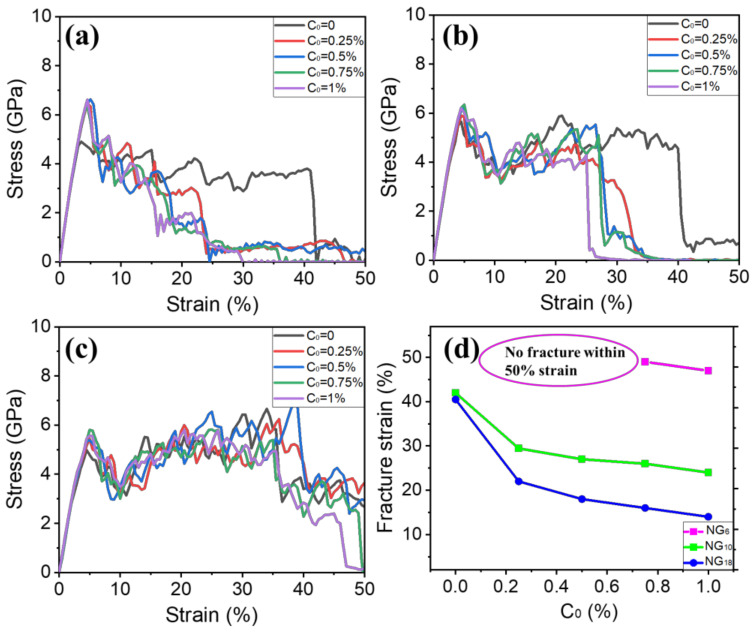
(**a**–**c**) Tensile stress–strain curves of NG_18_, NG_10_ and NG_6_ models, respectively, with different bulk H concentrations; (**d**) fracture strain as a function of C_0_.

**Figure 6:**
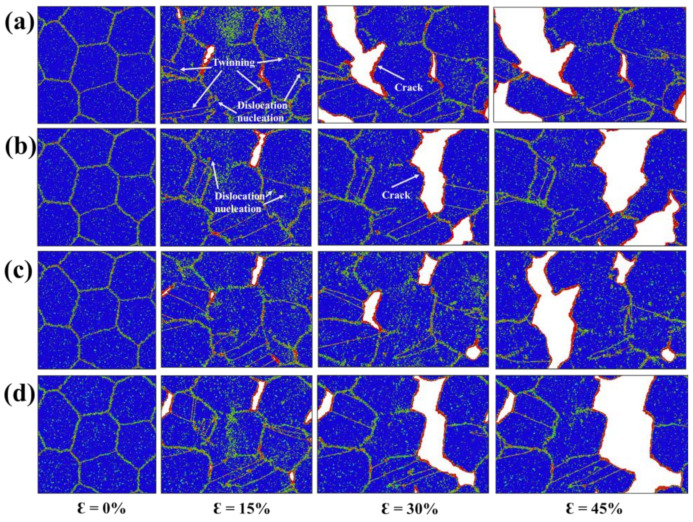
(**a**–**d**) The atomic snapshots of NG_18_ at 0%, 15%, 30% and 45% tensile strains with bulk H concentrations of 0%, 0.25%, 0.5%, 0.75% and 1%, respectively.

**Figure 7:**
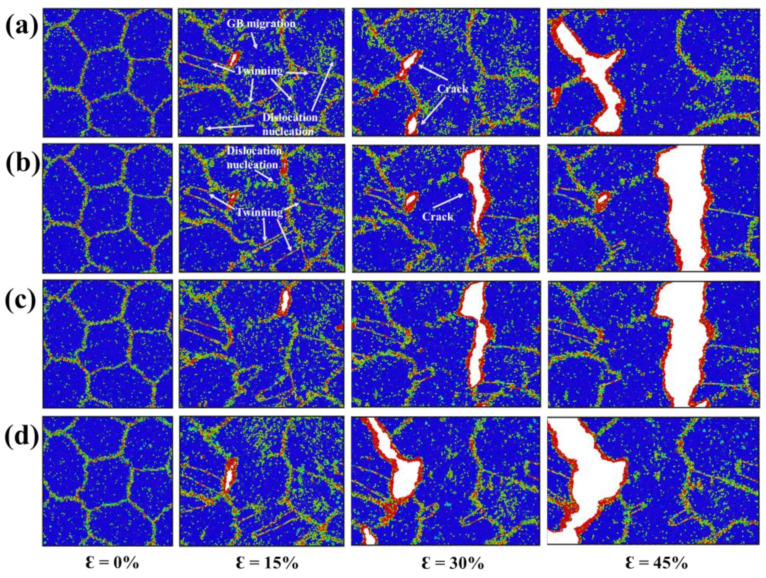
(**a**–**d**) The atomic snapshots of NG_10_ at 0%, 15%, 30% and 45% tensile strains with bulk H concentrations of 0%, 0.25%, 0.5%, 0.75% and 1%, respectively.

**Figure 8:**
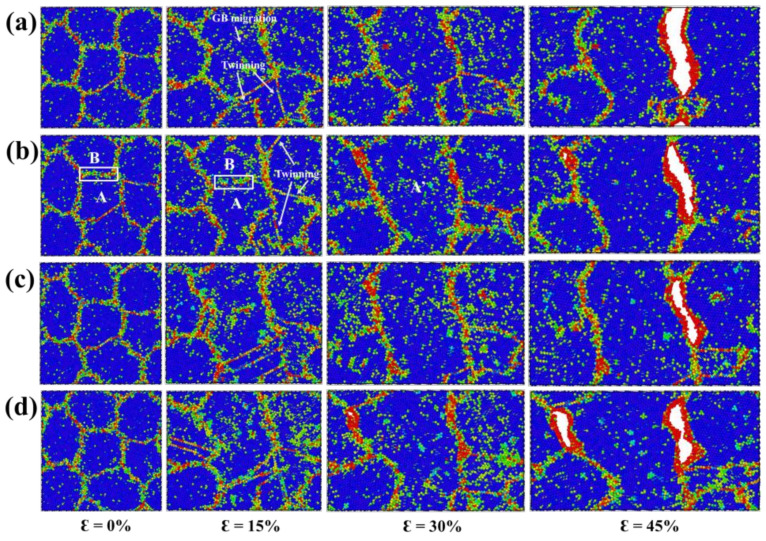
(**a**–**d**) The atomic snapshots of NG_6_ at 0%, 15%, 30% and 45% tensile strains with bulk H concentrations of 0%, 0.25%, 0.5%, 0.75% and 1%, respectively.

Uniaxial tension was carried out for each nanograined Fe model along the x direction with various bulk H concentrations considered for compassion. The tensile stress–strain curves of nanograined Fe models with five bulk H concentrations are plotted in Figure 5. It is obvious that the yield stress was higher when the bulk H concentration increased, which is indicative of the fact that H atoms impede the onset of plastic deformation. The embrittling effect that stems from H atoms can be observed, as the fracture strain was reduced with increases in the bulk H concentration. Furthermore, it was found that the susceptibility of nanograined Fe to intergranular embrittlement due to H was dependent on the grain size. In order to reveal these H-related deformations and fractures, the atomic configurations of the corresponding models with various bulk H concentrations are elaborated in Figure 6, Figure 7 and Figure 8.

The atomic configurations of NG_18_ at 0%, 15%, 30% and 45% tensile strains with bulk H concentrations of 0%, 0.25%, 0.5%, 0.75% and 1% are presented in Figure 6. At low H concentrations, the plastic process occurred by means of the nucleation of dislocations from the grain interior and GBs. Some twins were also observed across the grain interior, which suggests an intragranular mode rather than intergranular mode. The failure feature was ductile as the fracture surfaces were curved. With the increase in the H concentration, the nucleation of dislocations and twins was suppressed, accompanied by a remarkable loss in ductility. This is direct evidence that H atoms increase the yield stress of NG_18_. At a strain of 15%, there were more activated cracks with 0.5% and 1% H atoms than that with 0% and 0.25% H atoms. These cracks advanced along the boundary plane in a brittle manner with further applied strain, leading to an ultimate failure. Such embrittlement is in accordance with the HEDE mechanism [23,24,25,26], where the cohesive strength of GBs is significantly weakened, and GB fracture is promoted.

The atomic configurations of NG_10_ at 0%, 15%, 30% and 45% tensile strains with bulk H concentrations of 0%, 0.25%, 0.5%, 0.75% and 1% are presented in Figure 7. It can be observed that dislocation nucleation events occurred during the deformation process. Moreover, GB-mediated deformation involving GB migration, GB sliding, grain rotation and growth was more prominent in NG_10_ than that in NG_18_. With the increase in the H concentration, the nucleation of dislocations and twins was suppressed, and GB-mediated deformation was also hindered. It is clear that the GB migration event was reduced in the presence of H. The presence of H severely destroyed the local boundary structures, a phenomenon discussed in our previous study. In terms of 〈100〉 mode GB, H atoms enhanced the dislocation interactions by changing the GB dislocation configurations, thereby suppressing the collective gliding of GB dislocations and consequent GB migration. Regarding 〈111〉 mode GB, H atoms disordered local boundary structures, and thus inhibited the GB structural transformation and changed the coupling mode [61]. Such H-impeded coupled GB motion increases the yield stress, as shown in Figure 5. In terms of failure mode, the fracture surfaces were composed of more disordered atoms, and were more curved than those in NG_18_, suggesting that small-grain models are more resistant to HE.

The atomic configurations of NG_6_ at 0%, 15%, 30% and 45% tensile strains with bulk H concentrations of 0%, 0.25%, 0.5%, 0.75% and 1% are presented in Figure 8. The segregated H atoms prevented the GB-related intergranular deformation. With increases in the H concentration, grains kept their initial shapes, while GB migration, GB sliding and grain rotation decreased. The nucleation of dislocations was also inhibited by the segregated H atoms, as few dislocation plasticity events were found at the stage of deformation. Note that despite the dragging effect of solute H on GB mobility, the normal GB displacement can still occur at a higher tensile strain. For example, the boundary between grain A and B was pinned by segregated H atoms at a strain of 15%, but migrated at the strain of 30%, leading to grain A evolving into grain A’ at the expense of grain B, as shown in Figure 8b.

By comparing with NG_18_ and NG_10_ models, it is clear that NG_6_ was the most resistant to the HE. The NG_6_ model featured the fracture surfaces that were composed of very disordered atoms with higher CSP values. Besides, the formed cracks could evolve into the ultimate failure, contrary to the flat fracture surfaces of the NG_18_ and NG_10_ models. This phenomenon can be attributed to two aspects. One is that the NG_6_ model had a higher GB density, which contributed to the GB-related intergranular deformation. Such intergranular deformation relieved the accumulated stress around GBs and thus inhibited fracture process. The other is associated with local H concentration at GBs. As tabulated in Table 1, the C_H-GB_ of small-grain models was lower than that of large-grain ones, evidenced by that the C_H-GB_ of NG_6_ was only half of that of NG_18_. With a lower C_H-GB_, the embrittling effect of H atoms on NG_6_ model was mitigated.

## 4. Discussion

The simulations show that there was a strong dependence of H segregation, H-related deformation and fracture on the grain size of nanograined Fe models. The local H concentration at GBs was reduced with the decrease in the grain size at a given bulk H concentration. H segregation increased the yield stress of nanograined models and impeded the onset of plastic deformation. Furthermore, the susceptibility of nanograined Fe to HE decreased as the grain size decreased.

Previous studies have measured diffusible H content by the thermal desorption spectrum, finding that the ultrafine-grained specimen introduced the largest amount of diffusible H, but the local H content per unit surface area of GB was reduced with the decrease in the mean grain size [62,63]. These studies investigated H segregation properties on a micrometer scale but were not able to provide nanoscale H diffusion and distribution around the defects. The present study explains H segregation from the atomistic perspective. The trapping ability is dependent on the number of trapping sites at GBs; therefore, the H segregation ratio is higher in the small-grain models that have more possible trapping sites. However, due to the ‘dilution effect’ of the high GB density, the local H concentration at GBs of small-grain models is still lower than that of large-grain models.

Figure 5 shows the H-induced hardening effect, where H segregation increased the yield stress and impeded the onset of plasticity. When the grain size was above 10 nm, the normal Hall–Patch relationship operated. Dislocations were nucleated from the grain interior and GBs, and the intragranular dislocation mechanism was the dominant strength-controlling process. H atoms can produce hardness as a result of possible H-dislocation interactions. H concentrations generate the Cottrell atmospheres, leading to the decrease in dislocation movement and a dislocation pinning effect [64,65,66], or an increased slip planarity [16,67]. Our results confirm that H atoms suppress the nucleation of dislocations and twins, accompanied by a large amount of ductility loss. This is direct evidence that H atoms increase the yield stress. As the grain size was decreased below 10 nm, there was a transition of deformation mode into GB-mediated intergranular deformation involving GB migration, GB sliding, grain rotation and growth. Previous MD simulations have pointed out that H atoms hinder GB mobility by disordering local structures of GBs [61]. Keeping this in mind, H atoms increase the yield stress corresponding to GB mobility.

Besides the change in the mechanical behavior of nanograined Fe models after H segregation, it is important to consider the ultimate fracture in the presence of H. Figure 5d compares the fracture strain of different nanograined models with various H concentrations. The small-grain models were less susceptible to HE, e.g., there was no observed failure of NG_6_ with bulk H concentrations of 0%, 0.25% and 0.5%. Even though the H concentration reached 1%, the fracture strain was at a very high value (about 46%). Conversely, the NG_18_ model fractured at a low strain of 22% when the bulk H concentration was 0.25%, and fracture strain was further decreased with increases in the bulk H concentration. Bai et al. [62] showed that due to a high local H concentration, secondary cracks formed on the fracture surfaces of the H-charged specimens with coarser grain sizes, which resulted in the earlier fracture. Figure 6, Figure 7 and Figure 8 reveal this point as there were more formed cracks along GBs of NG_18_ due to a high local H concentration, and an easier brittle fracture compared to NG_6_. Apart from this, the GB-mediated intergranular deformation of small-grain models might also be resistant to HE. Solute H atoms are detached and left behind from GBs during GB migration process, consequently the cohesive strength of GBs cannot be weakened by solute H, and HEDE cannot operate [61]. These results clearly indicate one possible pathway by utilizing grain refinement to resist H-induced brittle failure, which is expected to prevent the HE problem of H storage and transportation systems and promote H energy used in transportation, energy storage and other fields.

## 5. Conclusions

In the present study, the effect of grain size on the HE behavior of nanograined Fe materials was investigated by MD simulations. It was found that the H segregation ratio increased, but local H concentration at GBs decreased with decreases in the grain size. When the grain size was above 10 nm, the intragranular dislocation mechanism was the dominant strength-controlling process, while GB-mediated intergranular deformation operated with the grain size below 10 nm.

H segregation increased the yield stress of nanograined models irrespective of the deformation mode. Furthermore, the simulations showed that small-grain models had less local H concertation at GBs and formed cracks, thus inhibiting ultimate fracture. These results are a clear indication of the utility of grain refinement to resist H-induced brittle failure.

## Figures and Tables

**Table 1:** GB characters and H segregation properties in NG_6_, NG_10_, NG_14_ and NG_18_.

Grain Size (nm)	$\Delta V_{\mathrm{GB}}$ (%)	GB Atom Ratio (%)	f_seg_, C_0_ = 0.5%	f_seg_, C_0_ = 1%	C_H-GB_, C_0_ = 0.5%	C_H-GB_, C_0_ = 1%
6	0.63	14.71	0.862	0.860	0.012	0.023
10	0.39	8.60	0.841	0.837	0.017	0.035
14	0.28	6.42	0.835	0.821	0.023	0.048
18	0.23	5.51	0.785	0.780	0.025	0.052

## Data Availability

The data presented in this study are available on request from the corresponding author.

## Nomenclature

HE	hydrogen embrittlement
GBs	grain boundaries
HEDE	hydrogen-enhanced decohesion
HELP	hydrogen enhanced localized plasticity
SPD	severe plastic deformation
HPT	high pressure torsion
ARB	accumulative roll bonding
ECAP	equal channel angular pressing
DPD	dynamic plastic deformation
MD	molecular dynamics
LAMMPS	large-scale atomic/molecular massively parallel simulator
OVITO	open visualization tool
CNA	common neighbor analysis
CSP	centro-symmetry parameter
DXA	dislocation extraction algorithm
NPT	Isothermal–isobaric ensemble
MSD	mean-squared displacement
